# The alterations of circulating mucosal-associated invariant T cells in polycystic ovary syndrome

**DOI:** 10.3389/fendo.2022.1038184

**Published:** 2022-11-28

**Authors:** Hong Zhou, Junting Xu, Ling Hong, Yanping Jia, Lilo Valerie Burk, Fengli Chi, Mei Zhao, Xiaohong Guan, Dan Liu, Xiangjie Yin, Yiqiao Zhang, Xiaoming Teng, Liyan Duan, Kunming Li

**Affiliations:** ^1^ Center for Assisted Reproduction, Shanghai First Maternity and Infant Hospital, School of Medicine, Tongji University, Shanghai, China; ^2^ Department of Gynecology and Obstetrics, University of Duisburg-Essen, Essen, Germany; ^3^ Department of Obstetrics and Gynecology, Shanghai First Maternity and Infant Hospital, School of Medicine, Tongji University, Shanghai, China; ^4^ Institute of Pharmacology, University Duisburg-Essen, Essen, Germany; ^5^ Reproductive Medicine Center, Nanjing Drum Tower Hospital, Nanjing University School of Medicine, Nanjing, China

**Keywords:** Mucosal-associated invariant T cells, Polycystic Ovary Syndrome, Interleukin 17, Interleukin 22, CD4+ Mucosal-associated invariant T cells

## Abstract

**Background:**

Polycystic ovary syndrome (PCOS) is the most common endocrine disorder affecting reproductive age females and an important cause of infertility. Although the etiology is complex and its pathogenesis remains unclear, the pathological process of PCOS is tightly related with the immune dysfunction and gut microbial dysbiosis. Mucosal-associated invariant T (MAIT) cells are a subset of innate-like T cells which can regulate inflammation through the production of cytokines and play a role in regulating the gut microbiota. We aim to evaluate the correlation between characteristics of PCOS and MAIT cells as well as their impact on cytokine secretion.

**Methods:**

Peripheral blood samples were taken from PCOS patients (n=33) and healthy controls (n=30) during 2-5 days of the menstrual period. The frequencies of MAIT cells and T cells were measured by flow cytometry. Cytokines interleukin 17 (IL-17), interleukin 22(IL-22), interferon γ (IFN-γ) and granzyme B were determined by Enzyme-linked immunosorbent assay (ELISA).

**Results:**

The frequency of MAIT cells was significantly reduced in the blood of PCOS patients compared with the controls, and negatively correlated with Body Mass Index (BMI), Homeostatic model assessment- insulin resistance (HOMA-IR) index, and Anti Miillerian Hormone (AMH). Thus, the frequencies of MAIT cells decreased in PCOS patients with abnormal weight (BMI≥24kg/m2), higher HOMA-IR (≥1.5), and excessive AMH (≥8ng/ml). The Cytokine IL-17 was significantly higher in PCOS patients and negatively correlated with the frequency of MAIT cells. Even though the IL-22 was lower in PCOS Patients, no correlation with MAIT cells was detected. In subgroup, CD4+MAIT cells correlated with BMI, AMH, and testosterone (T) levels.

**Conclusion:**

The frequency change of MAIT cells may play a significant role in the pathogenesis of PCOS. Exploring these interactions with MAIT cells may provide a new target for PCOS treatment and prevention.

## Introduction

Polycystic ovary syndrome (PCOS) is the most common reproductive endocrine disorder affecting reproductive age females, with an incidence of 5% to 20% worldwide (1). A Chinese community population study found the prevalence of PCOS in China is 5.6% (2).

PCOS has heterogeneous clinical characteristics: polycystic ovarian morphology (PCOM), clinical or biochemical hyperandrogenism, anovulation, and metabolic disorders such as insulin resistance (IR) and obesity (3–5). In the long term, patients with PCOS are at increased risk of diabetes, cardiovascular diseases, endometrial cancer, pregnancy complications and depression due to metabolic disturbance, which result in a heavy global burden (6–10). Although the etiology of PCOS has been studied for decades, the pathogenesis is still unclear and a causative therapy is still missing. Treatments are currently tailored to specific symptoms, include oral contraceptives or hormones to manage the menstrual cycle and control the symptoms of hyperandrogenemia, lifestyle modifications and metformin to improve metabolic syndrome such as insulin sensitivity and obesity, ovulation induction and assisted reproductive techniques to manage the PCOS-induced infertility (11).

Recently, researchers have focused on the hormones and immune cells, including both innate and adaptive immune cells, which have been reported to be a cross-talk in PCOS (12–14). It is well known that the endocrine markers of PCOS are hyperandrogenism and hyperinsulinemia. Previous studies reported a relatively high leukocyte count in PCOS patients with hyperinsulinemia and hyperandrogenemia, which exhibits a low-grade chronic inflammatory state and accounts for a disturbance in T cell polarization (15, 16). T cell polarization markers, such as interferon γ (IFN-γ) and interleukin 17 (IL-17) were increased in PCOS patients (17). However, interleukin 22(IL-22) secreted by innate lymphocytes leading to an improved IR was decreased in PCOS patients (18). Further results showed that granzyme-B was higher in PCOS patients which was positively correlated with hyperandrogenemia (19).

Mucosal-associated invariant T (MAIT) cells are a kind of unconventional innate-like T cells defined as CD3+CD161+Vα7.2+cells. They express semi-constant T cell receptor (TCR) composed of TCRα and TCRβ chains (20, 21). This receptor can specifically identify microbial-derived vitamin B metabolites presented by major histocompatibility complex class I-related protein 1(MR1) and plays a crucial role in local and systemic immune-inflammatory responses (22, 23).

MAIT cells are abundant in humans and comprise 1-10% of blood CD3+T cells. They are more enriched in the liver, intestine, and other mucosal tissues (23). Furthermore MAIT cells play a key role in the immune system and other pathologies such as tumors, they can attack healthy cells and thus contribute in certain autoimmune diseases and microbial infection (24–32). Previous studies found that alterations in the frequency and function of MAIT cells in circulation are associated with many metabolic diseases, such as diabetes/IR and obesity (33–37). As we know, PCOS is tightly associated with metabolic disorders, therefor we hypothesize that MAIT cells are related to the disorder of PCOS patients.

Until now, there are five different MAIT subsets which are CD4+CD8-, CD4+CD8+, CD4- CD8-, CD4- CD8αα+ and CD4- CD8αβ+ subset (38, 39). About 95% MAIT cells are CD4- subset (CD4– CD8αα+ or CD4– CD8αβ+ MAIT cells accounts for 80%, CD4- CD8- MAIT constitute 15%), only 5% of MAIT cells are CD4+ MAIT (CD4+CD8- and CD4+CD8+ subsets of MAIT cells) (39). Whereas CD4+ MAIT were focused on less, as this subset may be functionally distinct from others, Zhu et al. found that CD8+ MAIT cells are associated with metabolic dysfunction in PCOS patients (40). To date there has been no research on the role of total MAIT cells in PCOS patients.

We analyzed the frequency and phenotype of circulating MAIT cells (total MAIT cells and CD4+MAIT cells) in the blood of PCOS patients to explore their plausible clinical relevance which may provide novel therapeutic strategies to interfere with the PCOS disease.

## Materials and methods

### Patient samples

Ethical approval for this study was granted by the Scientific and Ethical Committee of the Shanghai First Maternity and Infant Hospital affiliated with Tongji University (NO: KS2132). All participating patients signed the informed consent. This study included 33 patients with PCOS and 30 healthy controls. The participants of control group were undergoing *in vitro* fertilization (IVF) or intracytoplasmic sperm injection (ICSI) due to male factor infertility or tubal factors, and PCOS patients were enrolled from the Endocrinology clinic or undergoing IVF or ICSI diagnosed according to the Rotterdam diagnostic criteria of 2003 (8) at Shanghai First Maternity and Infant Hospital between December 2020 and February 2022. All participants were between 18 to 40 years old, had no history of hereditary or familial diseases, and did not take any drugs. Patients with thyroid dysfunction, diabetes, other endocrine disorders, endometriosis, and autoimmune diseases were excluded. All patients’ clinical data are listed in **Table 1**.

**Table 1:** Clinical and biological characteristics of PCOS patients and healthy controls.

Patients	Control (n=30)	PCOS (n=33)	P-value
Age, (year)	29.83 ± 4.53	29.45 ± 5.25	0.761
BMI, (kg/m^2^)	20.90 ± 2.87	23.88 ± 2.81	*<0.001*
T, (ng/ml)	0.27 ± 0.08	0.39 ± 0.12	*<0.001*
AMH, (ng/ml)	4.23 ± 2.90	11.14 ± 5.03	*<0.001*
Basal FSH, (IU/L)	6.35 ± 1.46	6.27 ± 1.27	0.821
Basal LH, (IU/L)	4.54 ± 2.31	10.87 ± 6.42	*<0.001*
LH/FSH	0.74 ± 0.34	1.77 ± 1.14	*<0.001*
Basal E2, (pg/ml)	46.78 ± 24.22	58.42 ± 25.42	0.094
FPG, (mmol/L)	4.54 ± 0.40	4.64 ± 0.38	0.331
FINS, (pmol/L)	6.69 ± 1.85	10.35 ± 5.18	*0.001*
HOMA-IR	1.36 ± 0.42	2.09 ± 1.06	*0.001*
TC, (mmol/L)	4.61 ± 0.78	4.55 ± 0.81	0.827
TG, (mmol/L)	0.84 ± 0.41	0.89 ± 0.33	0.616

### Flow cytometry analysis

100ul of whole blood was incubated for 2o minutes at room temperature in the dark with the following antibodies: anti-CD3 PE (317308, BioLegend, San Diego, USA), anti-CD161 PE-Cyanine 7(339918, BioLegend, San Diego, USA), anti-TCR Vα7.2 APC(351708, BioLegend, San Diego, USA), and anti-CD4 FITC(300505, BioLegend, San Diego, USA), incubated at room temperature, in the dark for 20 minutes. Cell lysis and fixation was performed with 1x RBC Lysis/Fixation Solution (422401, BioLegend, San Diego, USA) for 15 minutes at room temperature in the dark. After centrifuging at 350g for 5 minutes, supernatant was discarded, then washed twice with Cell Staining Buffer. The cell pellet was resuspended in 500µl Cell Staining Buffer (420201, BioLegend, San Diego, USA) and analyzed on BD FACSCalibur flow cytometer using the CellQuestPro Software (BD Biosciences). Data were analyzed using FlowJo Version 10.5.3 software (TreeStar). The serum obtained after centrifugation was stored at -80°C.

### Enzyme-linked immunosorbent assay

Serum IL-17(EH6264M, Weiao, Shanghai, China), IL-22(EH6285M, Weiao, Shanghai, China), IFN-γ (EH6242M, Weiao, Shanghai, China), and granzyme B (EH6217M, Weiao, Shanghai, China) concentrations were measured using commercial ELISA kits according to the manufacturer’s protocol and procedure, each serum sample and assay diluent were placed in each well of a 96-well plate coated with IL-17/IL-22/IFN-γ/granzyme B antibody, then incubated for 30 minutes in a 37°C incubator, and each well was washed five times with wash solution, Subsequently, SABC-complexes was added into each well and the reaction was performed for 20 minutes in a 37°C incubator. Again, each well was washed five times with wash solution, after which a TAB reagent was added for 10 minutes in a 37°C incubator. The reaction was quenched by the addition of stop solution. Within 15 minutes, the absorbance was measured at 450 nm using a multifunctional microplate reader. The serum concentration was determined based on a standard concentration curve.

### Statistical analysis

All statistical analysis was performed using GraphPad Prism version 9.1.0 or OSX (GraphPad Software, La Jolla, CA). Results are expressed as mean ± SD. Groups were compared using the Student t-test or Mann–Whitney U test. Individuals were compared using paired t-test when applicable. Parameter correlation was determined using Pearson correlation coefficients. Levels of significance are indicated as *p < 0.05 or **p < 0.01.

## Result

### Clinical characteristics of patients

33 PCOS patients and 30 age-matched healthy controls were enrolled in the Shanghai First Maternity and Infant hospital from November 2020 to February 2022. **Table 1** shows the main clinical biological characteristics of PCOS and healthy controls included in the study. There were no significant differences in age, basal Follicle-Stimulating Hormone (FSH), estrogen (E2) fasting glucose (FPG), total cholesterol (TC), and triglycerides (TG). While the indicators of total testosterone(T), AMH, Luteinizing Hormone (LH), LH/FSH, Body Mass Index (BMI), fasting insulin (FINS), and HOMA-IR were higher in PCOS patients compared with the healthy controls.

### Decreased MAIT cells in peripheral blood CD3+T cells of PCOS patients

**Figure 1:**
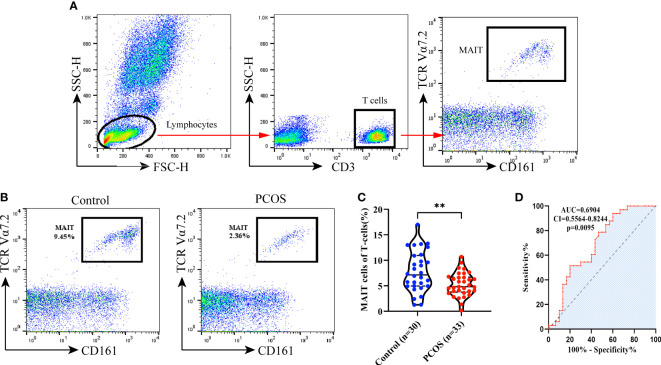
Frequency of MAIT cells in controls and PCOS patients. **(A)** Gating strategy for identification of circulating MAIT cells (CD3+ TCRVα7.2+CD161+). **(B)** Representative flow plots show the MAIT cells gated among CD3+ T cells in control and PCOS patients. **(C)** The frequency of MAIT cells in PCOS patients (n = 33) was significantly decreased compared to control (n = 30) (p<0.01). Data represent means ± SEM, **p < 0.01, significantly downregulated compared to control which analyzed by Mann–Whitney U test. **(D)** ROC curves of the MAIT cells in the identification of the PCOS patients (the area under the curve (AUC), confidence interval (CI), and the associated calculated P-value (p) are indicated on each graph).

Previous studies have suggested that CD3+CD161+TCR Vα7.2+ T cells can be considered MAIT cells (40, 41). Accordingly, the frequency of circulating CD3+CD161+ TCRVα7.2+cells in total CD3+ lymphocytes in individual subjects was determined by flow cytometry (**Figure 1A**). We first analyzed the frequency of circulating CD3+Vα7.2+CD161+ MAIT cells, the percentages of circulating MAIT cells were significantly lower in PCOS patients than in control (5.29 vs. 7.76%, p<0.01, **Figures 1B, C**). Concerning the diagnostic accuracy of MAIT cells frequency for PCOS, the ROC curve analysis of MAIT cells in PCOS patients, the AUC was 0.69 (95% CI 0.56–0.82, P <0.05) (**Figure 1D**). Therefore, PCOS patients had significantly reduced numbers of circulating MAIT cells.

### Correlation between MAIT cells clinical characteristics in PCOS patients

**Figure 2:**
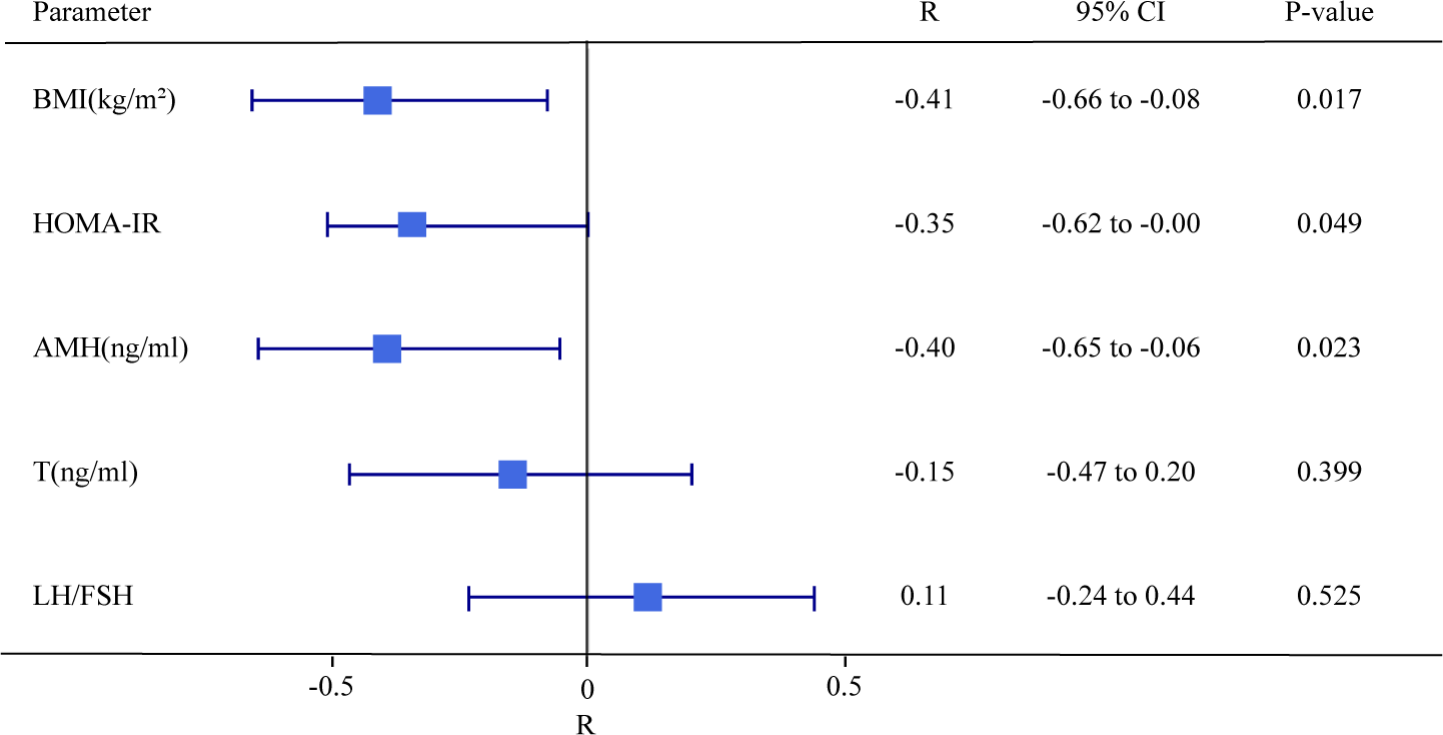
Forest plot of the Pearson Correlation between frequencies of MAIT cells of T cells and BMI, HOMI-IR, AMH, T, and LH/FSH in PCOS patients. P < 0.05 is considered as significantly different.

Multiple regression analysis was performed to analyze the correlation of BMI, HOMA-IR index, AMH, T, LH/FSH, and MAIT cells frequency. As shown in **Figure 2**, the frequency of MAIT cells was significantly negatively correlated with BMI, HOMA-IR index, and AMH (R=-0.41, -0.35, -0.4; p=0.0017, 0.049, 0.023), and whereas no significant correlation between MAIT cells and LH/FSH or T could be found.

### Analysis of MAIT cells in different subgroup of PCOS patients

**Figure 3:**
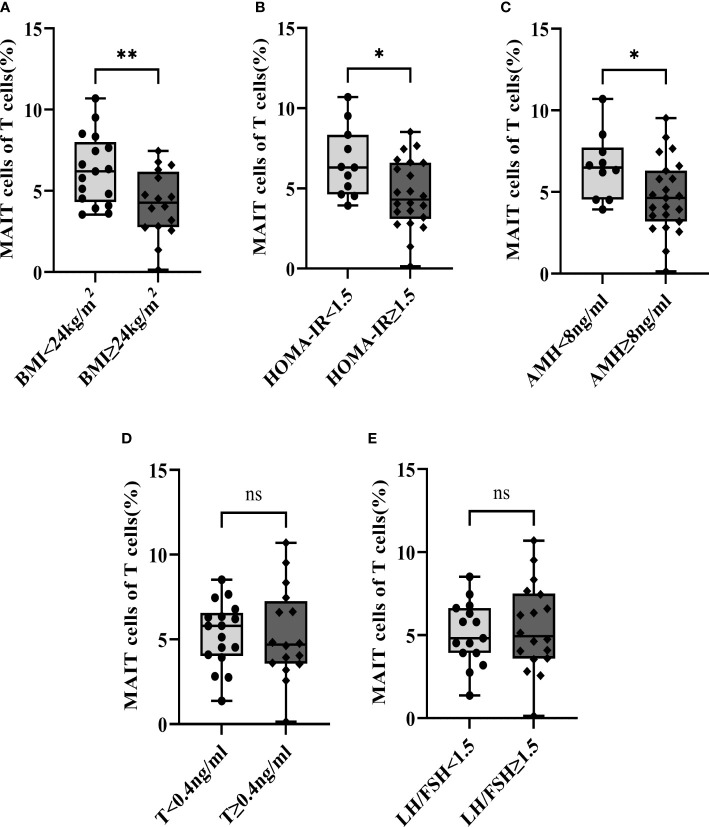
Subgroup analysis of the frequencies of MAIT cells of T cells in PCOS patients by BMI, HOMA-IR, AMH, T, and LH/FSH. **(A)** BMI; **(B)** HOMA-IR; **(C)** AMH; **(D)** T; **(E)** LH/FSH. *, p<0.05, **, p<0.01, ns, not significant.

Due to the frequency of MAIT cells being significantly negatively correlated with BMI, HOMA-IR index, and AMH, 33 PCOS patients were divided into different subgroups by BMI, HOMA-IR, AMH, T, and LH/FSH. We found that the frequencies of MAIT cells of T cells decreased in PCOS patients with abnormal weight [BMI≥24kg/m2 (41)] (**Figure 3A**), higher HOMA-IR (≥1.5) (42, 43) (**Figure 3B**), and excessive AMH [≥8ng/ml (44)] (**Figure 3C**). However, T and LH/FSH do not affect the frequencies of MAIT cells (**Figures 3D, E**).

### CD4+ MAIT cells

**Figure 4:**
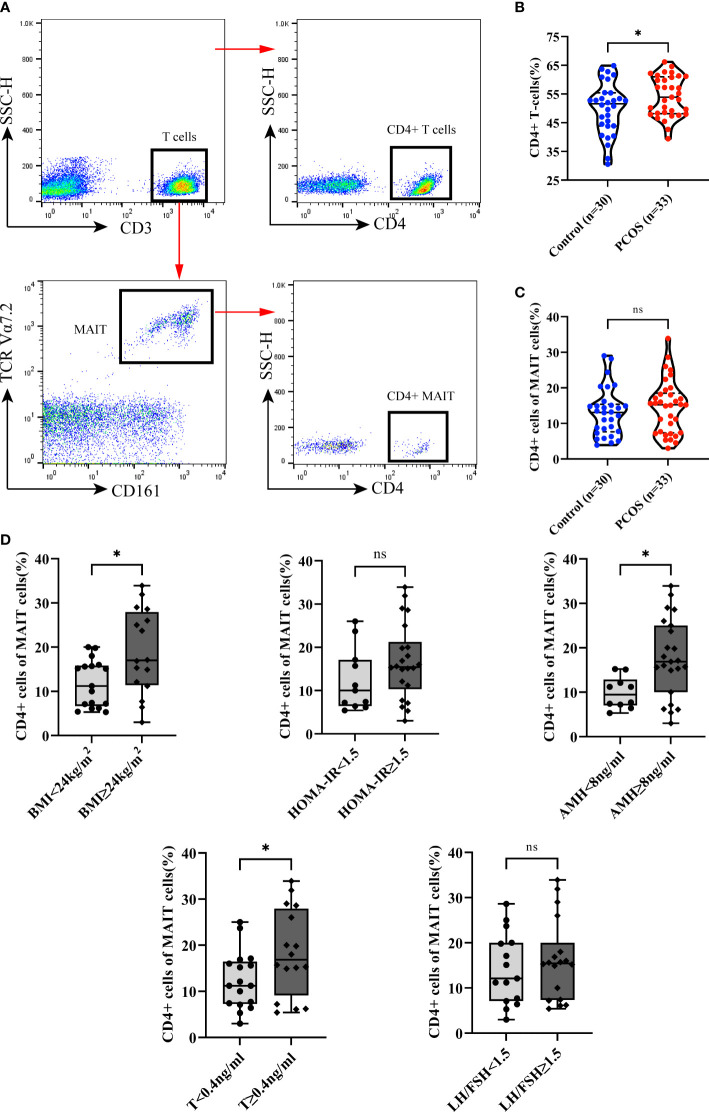
CD4+ cells of MAIT cells and T cells between Control and PCOS groups, and subgroup analysis of the CD4+ cells of MAIT cells in PCOS patients by BMI, HOMA-IR, AMH, T, and LH/FSH. **(A)** Gating strategy for CD4+ T cells (CD3+ CD4+) and CD4 MAIT cells (CD3+TCRVα7.2+CD161+CD4+). **(B)** CD4+ cells of T cells between Control and PCOS groups. **(C)** CD4+ cells of MAIT cells between Control and PCOS groups. **(D)** Subgroup analysis the CD4+ cells of MAIT cells in PCOS patients by BMI, HOMA-IR, AMH, T, and LH/FSH. *p<0.05, ns, not significant.

Although only 5% of MAIT cells are CD4+ MAIT (CD4+CD8- and CD4+CD8+ subsets of MAIT cells) (39), they may have a distinct role in the immune system. However, a small proportion of CD4+MAIT cells were found in PCOS patients and healthy control group, there was no significant difference in CD4+MAIT cells between the two group (**Figures 4A, C**). then we analysis the CD4+ cells of T cells, we found that the CD4+ T cells increased in PCOS patients (**Figures 4A, B**). Interestingly, we found CD4+MAIT cells significantly increased in PCOS patients with abnormal weight (BMI≥24kg/m2), excessive AMH (≥8ng/ml), and excessive T (≥0.4ng/ml). However, CD4+MAIT cells did not show any difference when we divide the subgroups according to HOMA-IR and LH/FSH (**Figure 4D**).

### Correlation between circulating MAIT cells of T cells and cytokines in plasma

**Figure 5:**
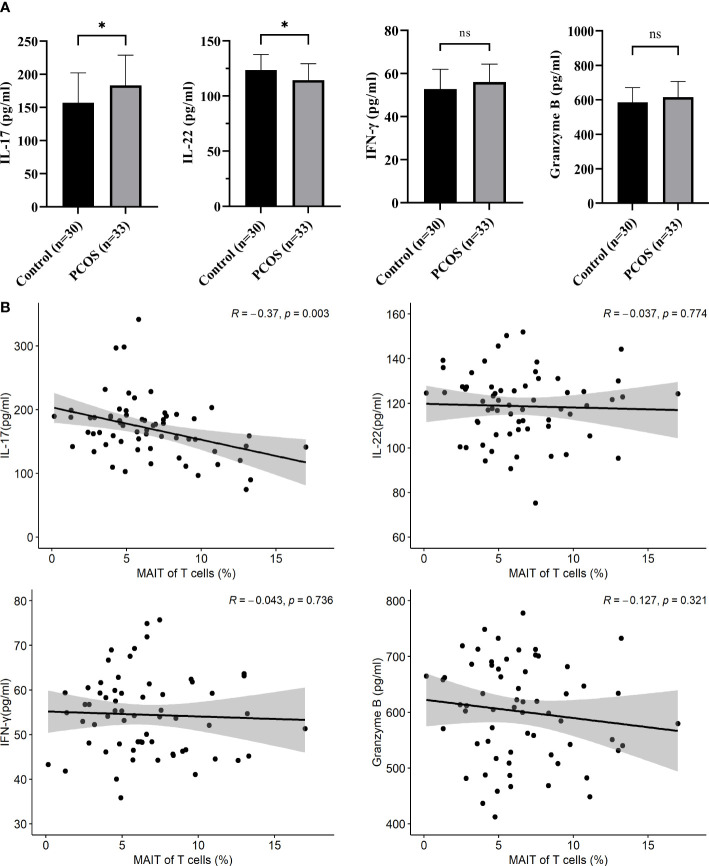
Cytokines in the plasma of control and PCOS patients. **(A)** compared L-17, IL-22, IFN-γ and granzyme B between control and PCOS patients. **(B)** The Pearson Correlation between the frequency of MAIT cells and cytokines in plasma. *p<0.05, ns, not significant.

MAIT cells can quickly respond to stimulation, which produce a range of cytokines (22). To better demonstrate the function of MAIT cells, we analyzed the relationship between MAIT cells and cytokines in PCOS, we evaluated the level of IL-17, IL-22, IFN-γ, and granzyme B in the plasma of all participants. IL-17 is higher in the plasma of PCOS patients while the IL-22 is less expressed in PCOS patients than in the control group. There were no significant differences in IFN-γ and granzyme B (**Figure 5A**). In addition, a negative relation between the level of IL-17 in plasma and the frequency of MAIT cells of T cells was found (**Figure 5B**).

## Discussion

This study revealed that the frequency of MAIT cells is decreased in peripheral blood of PCOS patients compared with control group. The decrease of MAIT cells indicates that MAIT cells are associated with the disorder of PCOS. Interestingly, we found that the frequency of MAIT cells were negatively correlated with the clinical features of BMI, HOMA-IR and AMH. It is known that AMH plasma level is a maker of impaired folliculogenesis in patients (45). Therefor we suggest that the altered distribution of MAIT cells can affect the development of PCOS *via* the aspects of metabolism and follicular development.

A previous study showed that blood MAIT cell frequency decreased in obesity and type-2 diabetes, even below the detection limit in severe obesity patients (46). MAIT cells in peripheral blood can be recruited to adipose which may contribute to the decrease MAIT cells number (46). It was reported that 3-6 months after bariatric surgery was performed in obese patients, the frequency of MAIT cells increased (46). As BMI increases, visceral adipose tissue (VAT) exhibits chronic low-grade inflammation, a major factor associated with IR (35). These results are consistent with our results that the frequency of MAIT cells were lower in overweight patients (BMI≥24kg/m^2^) and with higher HOMA-IR (≥1.5). Rouxel et al. reported that the frequency of MAIT cells in the peripheral blood of diabetic patients is lower than in healthy people, and this reduction in frequency may be due to the migration of MAIT cells into the pancreas of mice during the development of diabetes, which can also kill human β-cell line (47). Besides, the lower numbers of blood MAIT cells might also lead to cell exhaustion as a previous study has shown they carry a defective BCL2, CD25 and PD-1 expression causing persistent activation (47) (46). However, further studies are needed to verify that MAIT cells are exhausted in PCOS patients.

Our study also observed that the level of IL-17 in plasma increased in PCOS patients and negatively correlated with the frequency of MAIT cells of T cells, which is consistent with the studies on diabetes and obesity (33, 48). Kousei et al. reported that IL-17 can activate Angiotensin II Type 1 Receptor inducing induced IR (49), which may also be the cause of IR in PCOS patients. However, IL-22 was decreased in PCOS which is consistent with Qi et al, who reported that the IL-22 levels in serum and follicular fluid of PCOS patients were decreased (18). IL-22 can be produced by intestinal group 3 innate lymphoid cells (ILC3), and Qi et al. found that transplantation of stool microbiota from mice with PCOS into recipient mice resulted in increased disruption of ovarian function, IR, altered bile acid metabolism which decreased the secretion of IL-22 from intestinal-type-3 innate lymphoid cells (18). MAIT cells are a kind of innate-like T cell, which are found in the intestine, skin, oral and female genital mucosa. The decrease of IL-22 may have a certain relationship with the changes of MAIT cells. This, however, could not be proven by our study possibly due to the small number of cases, or the MAIT subtype. Therefore, further research is needed which include a large patient cohort longitudinal studies and more subsets of MAIT cells.

Although the CD4+ MAIT cells subset constitutes only a small fraction which usually were ignored for their low frequency, studies have shown that CD4+ MAIT cells can produce more IL-22 than other MAIT subset (38). Therefore CD4+MAIT cells may play a distinct role in disease. Studies that investigated the association between PCOS and IL-22, found that overweight PCOS patients had significantly higher levels of IL-22 in serum compared to healthy controls (14, 50).We speculate that elevated Il-22 may be associated with an increase in CD4+MAIT cells. In our study, we did not find a difference between the control and PCOS group, however the CD4+ T cells increased in PCOS patients, this is consistent with previous literature reports (51), that might be the reason for indifference of CD4+ MAIT between two group. In the subgroup, however, we found that the proportion of CD4+MAIT cells correlates with BMI, AMH, and testosterone levels. CD4+MAIT were higher in overweight patients (BMI≥24kg/m^2^), and patients with higher AMH(≥8ng/ml) and T (≥0.4ng/ml). AMH is regarded as the best serum biomarker of ovarian reserve. Previous studies revealed that total testosterone is closely related to AMH and plays an important role in follicular growth (52). A study in 2016 found that the number of circulating CD4+ cells strongly affected serum AMH levels (53). Therefore, CD4+MAIT cells may play a key role in ovarian granulosa cell function and follicular physiology.

## Conclusion

The outcomes of our study showed that the reduction of MAIT cells and increased frequency of CD4+MAIT cells may contribute to the metabolic disorder and follicular development in PCOS. MAIT cells may act as a predictive marker and could present potential new treatment options. However, the limitation of this study is that we only correlate the frequency to the clinical features, which support the hypothesis that MAIT cells and CD4+MAIT cells play a role in PCOS. Further in-depth research of the correlation between different MAIT cells subsets and PCOS will be performed.

## Data availability statement

The raw data supporting the conclusions of this article will be made available by the authors, without undue reservation.

## Ethics statement

The studies involving human participants were reviewed and approved by Shanghai First Maternity and Infant Hospital ethics committee (NO: KS2132). The patients/participants provided their written informed consent to participate in this study. Written informed consent was obtained from the individual(s) for the publication of any potentially identifiable images or data included in this article.

## Author contributions

KL, LD, HZ and JX contributed to study designed and interpretation of data. LD, and KL supervised the experiments. HZ and JX performed the experiments. YJ, XG, LB, DL, XY and YZ assisted with manuscript drafting and critical discussions. LH, FC, MZ and KL collect clinical information. All authors contribute to the article and approved the final manuscript.

## Funding

This work was supported by grants from the National Natural Science Foundation of China (Grant No.82201881), the Natural Science Foundation of Shanghai (Grant No.21ZR1450700), and the Medical Consortium of Shanghai Pudong (Grant No. PDYLT2022-14).

## Conflict of interest

The authors declare that the research was conducted in the absence of any commercial or financial relationships that could be construed as a potential conflict of interest.

## Publisher’s note

All claims expressed in this article are solely those of the authors and do not necessarily represent those of their affiliated organizations, or those of the publisher, the editors and the reviewers. Any product that may be evaluated in this article, or claim that may be made by its manufacturer, is not guaranteed or endorsed by the publisher.
